# Muscle Synergy of Lower Limb Motion in Subjects with and without Knee Pathology

**DOI:** 10.3390/diagnostics11081318

**Published:** 2021-07-22

**Authors:** Jingcheng Chen, Yining Sun, Shaoming Sun

**Affiliations:** 1 Institute of Intelligent Machines, Hefei Institutes of Physical Science, Chinese Academy of Sciences, Hefei 230031, China; cjc324@mail.ustc.edu.cn (J.C.); ynsun@iim.cas.cn (Y.S.); 2 University of Science and Technology of China, Hefei 230026, China

**Keywords:** knee pathology, lower limb motions, surface electromyography, muscle synergy analysis, non-negative matrix factorization, feature selection

## Abstract

Surface electromyography (sEMG) has great potential in investigating the neuromuscular mechanism for knee pathology. However, due to the complex nature of neural control in lower limb motions and the divergences in subjects’ health and habits, it is difficult to directly use the raw sEMG signals to establish a robust sEMG analysis system. To solve this, muscle synergy analysis based on non-negative matrix factorization (NMF) of sEMG is carried out in this manuscript. The similarities of muscle synergy of subjects with and without knee pathology performing three different lower limb motions are calculated. Based on that, we have designed a classification method for motion recognition and knee pathology diagnosis. First, raw sEMG segments are preprocessed and then decomposed to muscle synergy matrices by NMF. Then, a two-stage feature selection method is executed to reduce the dimension of feature sets extracted from aforementioned matrices. Finally, the random forest classifier is adopted to identify motions or diagnose knee pathology. The study was conducted on an open dataset of 11 healthy subjects and 11 patients. Results show that the NMF-based sEMG classifier can achieve good performance in lower limb motion recognition, and is also an attractive solution for clinical application of knee pathology diagnosis.

## 1. Introduction

Lower limb motions are essential in many human activities, especially the ability to independently perform the activity of daily living (ADL), such as standing, sitting and walking, and very important for avoiding injuries caused by falls, improving personal happiness and reducing the social costs of nursing. Recently, sensor-based intelligent motion recognition and disease diagnosis has been greatly developed due to the rapid development of sensing, machine learning and computing science. By analyzing the sensor data during motions, we can establish an objective method to identify a patient’s movement intention or disease state, and this has been widely applied in fall detection [1,2], artificial limbs and exoskeletons [3,4], as well as disease monitoring and assessment [5,6]. Traditional technologies for motion identification and disease assessment require expensive laboratorial instruments, such as gait channel, electro-optical motion capture and three-dimensional force platform. Compared with these methods, surface electromyography (sEMG) is a kind of portable, non-invasive and low-cost wearable device, which has been widely used for assessing human activities, especially in the field of neurological or musculoskeletal disorder diagnosis, exoskeleton control and rehabilitation guidance [7,8,9].

The sEMG sensors are used to record and analyze muscle activation during gestures and motions. Each voluntary motion involves the activation of a large amount of muscles, and the functions of muscles vary from motion to motion. For example, when performing leg flexion at standing position (STD), the gluteus maximus, quadriceps, triceps surae and erector spinae are recruited to keep balance for straight standing, while femoral biceps (FB) and semitendinosus (SEM) are recruited as agonistic muscles with rectus femoris (RF) and vastus medialis (VM) as antagonist muscles for knee flexion. Leg extension at sitting position (ST) involves recruitment of a powerful group of thigh muscles, which are quadriceps recruited as agonistic muscles and hamstrings recruited as antagonists. In addition, most of the lower limb muscles are recruited for multiple functions when performing complex and nature movements like gait exercise (Gait). STD, ST and Gait reveal the main functions and activation patterns of knee muscles. Therefore, study of the lower limb muscle activation pattern during the aforementioned motions can help us to investigate the effects of knee pathology on neuromuscular control during voluntary movement, and to establish objective metrics for disease diagnosis. Moreover, classification on patients’ lower limb motions is an important and challenging task in remote disease monitoring and rehabilitation based on task-specific assessment, as well as neural control of external devices to assist movements of patients with lower limb motor dysfunction or amputation. Such applications require the use of a compact and portable system while overcoming the classification bias caused by various muscle abnormalities. Thus, to design a robust lower limb motion classifier for subjects with and without knee pathology is one of the objectives of this work.

Note that the sEMG signals of thigh muscles are often multilayer overlapping of surface and deep muscles [10,11,12], and performing lower limb motions is often accompanied by a wide range of body movements and large load bearing, which leads to the complex nature of the signals. This problem is more pronounced in subjects with muscular abnormalities. As a matter of fact, not only for the lower limb motions, voluntary movements often involve multiple muscles and joints, which makes it difficult to extract robust and useful information from raw sEMG data. Thus, it is necessary to establish a method to explore the mechanism of motor nervous system on multi-freedom control. In order to explain this mechanism, Bernstein [13] proposed a hypothesis of muscle synergy. The muscle synergy hypothesis holds that the central nervous system (CNS) tends to achieve efficient and precise control of a multi-degree of freedom by combining a small amount of discrete muscle coordination patterns that are fixed and embedded in the brainstem and spinal cord. That is, in the framework of muscle synergy, one synergy can activate multiple muscles at a fixed intensity, while one muscle can be activated by multiple muscle synergies. Figuratively, muscles are like letters, muscle synergies are words, and voluntary movements are sentences composed of individual words [14]. 

Recent studies on the mechanism of motor neural control have provided evidence for the hypothesis of muscle synergy. For instance, researchers found that the muscle activation patterns that humans recruit for different sports (walking, jumping, swimming, kicking a ball) have similar synergy structures [15]. While studies on postural responses in newborns have shown that the muscle synergy pattern can be altered by learning on the one hand, and is also innate on the other [16], which explains the similarities and personalities between different people when performing the same movement. In addition, from the perspective of a biomechanical model, researchers also proved that the muscle synergy model can achieve a good reconstruction of complex motor behavior [17,18].

As muscle synergy patterns are the underlying mechanisms of the neuromuscular control system, it is difficult to obtain such information directly from external sensors. One solution is to perform matrix factorization algorithms on the raw sEMG signals, which can reveal complex connections between different channels, and isolate a linear combination of deep components, which are likely to be closely related to the motor unit action potential, from the source information [19]. Non-negative matrix factorization (NMF) provides a method to extract the linear representations of the input data which are a mixture of multiple channels of information [20,21]. Unlike other matrix factorization algorithms, such as principal component analysis (PCA) and independent component analysis (ICA), NMF requires that the sub-matrices obtained by decomposition be non-negative, which means the raw data can be described as an additive combination of lower-dimensional patterns [22]. Because all the muscle activation data are non-negative and there is no need to limit muscle synergy patterns to orthogonal to each other, therefore, NMF is more suitable for muscle synergy extraction than other matrix factorization algorithms.

Several works have been carried out to study the underlying mechanism of neuromuscular control for a variety of human activities based on the NMF-based muscle synergy analysis, such as crawling [23], walking [24], running [25], golfing [26] and dart throwing [27]. Furthermore, the function of this method to reveal intrinsic patterns in the neuromuscular system prompted the researchers to use it to study the expression of human muscle states. For example, Smale et al. [28] proposed a method to supplement continuous wavelet transforms with muscle synergies in the traditional sEMG fatigue analysis and achieve better performance of describing muscle fatigue. Studies on patients with Parkinson’s disease [29], cerebral palsy [30], and spinal cord tract disease [31] have found that the muscle synergy of these patients are different from those of healthy people when performing specific tasks, and NMF-based sEMG analysis can be used to establish assessment metrics for the diseases. In addition, in engineering application, Lin et al. [32] proposed a multi-degree of freedom control method for artificial limb based on NMF of sEMG with the inclusion of sparseness constraints. Experiments were conducted on normal subjects and two patients with unilateral limb defects, and achieved better performance than the previous supervised algorithms. Naik et al. [22] proposed a NMF-based method to classify 10 finger movements recorded from two sEMG sensors up to 92% accuracy. Similarly, a series of studies on upper limb motion based on NMF of sEMG have been carried out in a normal person and patients [33,34,35,36], which indicated that it is also feasible to use this method to study the lower limb motions.

The present study first extracts the thigh muscle synergy of subjects with and without knee pathology when performing three different lower limb motions, and then analyzes and compares the muscle synergy patterns and corresponding activation coefficients of different subjects during different motions. Therein, a muscle synergy similarity calculation method is proposed and a lower limb motion classifier based on the features extracted from muscle synergy matrices is designed. Moreover, a pilot study is conducted to explore the feasibility of the diagnosis of knee pathology with NMF of sEMG data. In particular, a two-stage feature selection method is introduced to investigate the best NMF-based feature sets for motion classification or knee pathology diagnosis.

## 2. Algorithm for Muscle Synergy Analysis

### 2.1. Muscle Synergy Extraction

**Figure 1:**
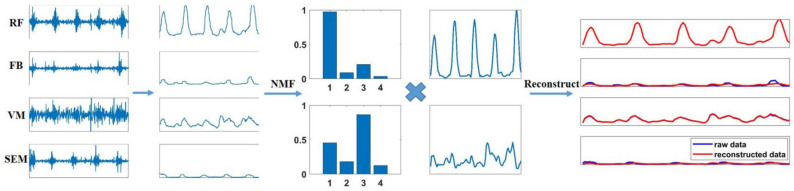
An example of the non-negative matrix factorization (NMF) decomposition of multiple channel surface electromyography (sEMG) signals. The muscles are the rectus femoris (RF), femoral biceps (FB), vastus medialis (VM) and semitendinosus (SEM). The five subgraphs from left to right are raw sEMG signals, envelops of the sEMG signals, the muscle synergy pattern when k = 2, the activation coefficient when k = 2 and the comparison diagram between the original envelops and reconstructed signals.

The NMF algorithm was used in this work to extract the muscle synergy pattern and the corresponding activation coefficient. Generally, a set of sEMG envelope signals obtained from multichannel recordings can be expressed as a m×n matrix $A_{m\times n}$, where *m* means the number of channels and *n* means number of samples. $A_{m\times n}$ is decomposed in two non-negative matrices *W* and *H* by NMF as:

(1) $$A_{m\times n}=W_{m\times k}\times H_{k\times n}+\varepsilon$$

where, $W_{m\times k}$ is the muscle synergy pattern matrix with *k* synergies, $H_{k\times n}$ is the activation coefficient matrix of the *k* synergies, and ε is the residue of the factorization. An example of NMF decomposition of multi-channel sEMG signals is shown in Figure 1.

The goal of NMF is to find *W* and *H* which solve:

(2) $$\begin{array}{cc} \mathrm{minimize} & J\left(A,W,H\right)\\ \mathrm{subject}\;\mathrm{to} & W\geq 0\;\mathrm{and}\;H\geq 0 \end{array},$$

where, $J\left(A,W,H\right)$ is the cost function and the one we used in this work is Euclidian distance, as:

(3) $$J=\left\|A-W\times H\right\|,$$

To solve the problem shown in Equation (2), a multiplicative update algorithm proposed by Lee and Seung [21] was applied due to its low computational complexity. The update rules are:

(4) $$\begin{array}{c} H\leftarrow H\times \frac{W^{T}\cdot A}{W^{T}\cdot W\cdot H}\\ W\leftarrow W\times \frac{A\cdot H^{T}}{W\cdot H\cdot H^{T}} \end{array},$$

For the proposed research, each column vectors of *W* in each iteration were normalized to make the synergy matrices of different subjects comparable. Moreover, to reduce the risk of falling into locally optimal solutions, the iterations were carried out 20 times begin with different random initial *W* and *H*, as well as one time with singular value decomposition (SVD) based initial *W* and *H* [37]. The final submatrices were determined as those with maximum variability accounted for (VAF), which is calculated as:

(5) $$VAF=1-\frac{{\left(A-W\times H\right)}^{2}}{{\left(A-\bar{A}\right)}^{2}},$$

Considering a special case in which the activation coefficient matrix is calculated when muscle synergy pattern matrix is determined. A modified version of Equation (4) was used, i.e., only the update algorithm of the *H* was used, while *W* remained unchanged.

### 2.2. Quantify the Similarity between Muscle Synergies

The muscle synergy consists of two parts, one is muscle synergy pattern and the other is activation coefficient. Similarity of different muscle synergy pattern matrices is calculated by the maximum correlation coefficient (CC) of the two matrices [30,38]. For two synergy matrices $W1_{m\times k1}$ and $W2_{m\times k2}$ (k1≤k2), the first step to calculate the maximum CC is to select *k*1 of the *k*2 column vectors of $W2_{m\times k2}$ and reconstruct them into a new matrix ${\bar{W2}}_{m\times k1}$, of which the CC with W1 can be easily obtained. The maximum CC is the largest CC of W1 with all the reconstructed matrices $\bar{W2}$. The intra-similarity of a group of muscle synergy pattern matrices is obtained by computing the average of the maximum CCs between all pairs of matrices within the group. The inter-similarity of two groups is figured out by averaging the maximum CCs between all pairs of matrices consisting of one matrix from each group. In addition, the method for quantifying similarity of the activation coefficient matrix is the same as that for the muscle synergy pattern matrix.

## 3. Materials and Methods

**Figure 2:**
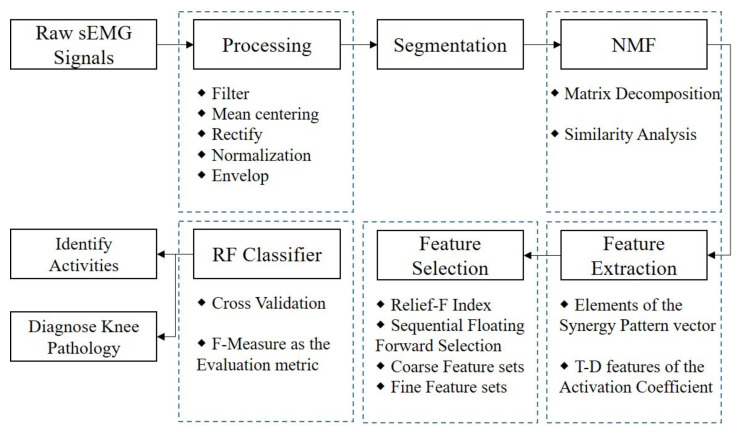
Schematic diagram of the NMF-based sEMG classifier for lower limb motions identification and knee pathology diagnosis.

The flow chart of methods in the present study is shown in Figure 2.

### 3.1. Data Description

Raw data were downloaded from the UCI Machine Learning Repository [39], which contains four channels of lower limb sEMG signals and one channel of goniometry signal obtained from 22 male subjects older than 18 years of age. Half of the subjects were healthy participants without any knee injury or pain (Control Group, CG), and the other half were individuals with knee pathology and had not started a rehabilitation process before the tests (Study Group, SG). Among the SG, six subjects had anterior cruciate ligament injury, four were suffering from meniscus injury and one’s sciatic nerve was injured. The raw data were recorded by using a data logger (Datalog MWX8 by Biometrics) with four channels of sEMG and one SG150B goniometer at sampling frequency of 1000 Hz. The surface electrodes were spaced apart by 20 mm and placed on the surface of four thigh muscles, which were RF, FB, VM and SEM. Meanwhile, the goniometer was attached to both sides of the knee to record the dynamics of knee angle during the experiments. All the sensors were attached to the left leg for normal subjects and to the diseased leg for abnormal subjects. During the experiments, each subject was asked to perform three lower limb motions, which were STD, ST and Gait, respectively. To be specific, STD was started with static standing, following with flexing the target shank slowly to achieve maximum flexion, keeping the flexion for a while and then extending the shank to the initial position. ST was started with sitting on a chair at rest, following with extending the target shank to the horizontal position, keeping the extension for a while and flexing the shank to the initial position at last. According to the description of Herrera-González [40], each subject was required to perform at least four times STD/ST according to the following time sequence: 2 s for flexing the leg, extending the leg and maintaining leg flexion/extension, respectively; while relaxing 3 s between each motion. The Gait was straight walking back and forth on level ground at the pace subject felt comfortable with. For each straight walking, subjects were required to take four steps on each leg.

### 3.2. Data Preprocessing

Data processing was performed on the Matlab software (MATLAB R2018a, The MathWorks, Inc., Novi, MI, USA). Raw sEMG data were first filtered through a fourth-order Butterworth 20–450 Hz bandpass filter, then zero-centered and rectified. Afterwards, the amplitude of the processed data of each muscle was normalized to the respective maximum amplitude across all movements [25]. Finally, a 100 ms moving average root-mean-square (RMS) window was used to extract the envelope of sEMG signals.

Moreover, the raw angle data were smoothed by using a moving average window, and then a segmentation algorithm was introduced to segment the movements. For ST and STD, the curve of knee angle in one motion is bell shaped, and the beginning point set to the first point reached 10% of the maximum speed over the extension/flexion phase in one movement for ST/STD, respectively, while the ending point was set to the first point less than 10% of the maximum speed over the flexion/extension phase. Meanwhile, the segment point for Gait was set to the first minimum point after the maximum joint angle, which was considered to be the beginning of the support phase.

**Figure 3:**
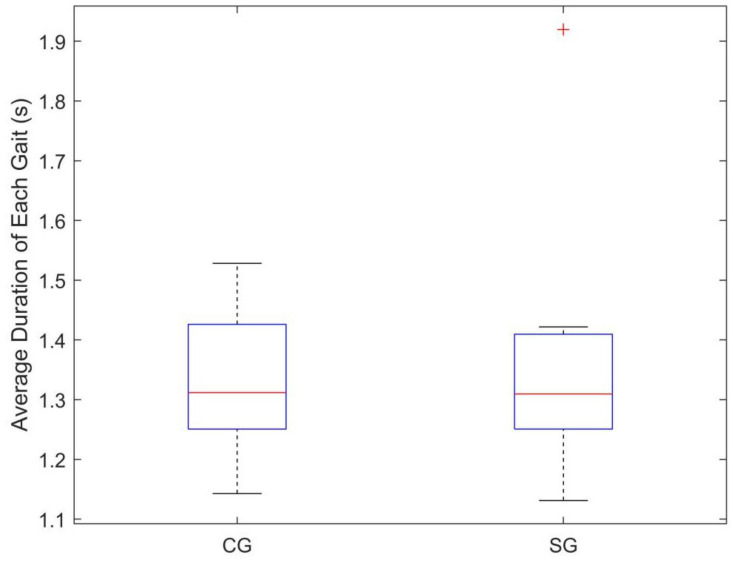
Average duration of each Gait for subjects in different groups. The ‘+’ in the figure represents the outlier.

Note that the walking speeds were different between subjects, which could induce variations. Although there is a lack of information about walking speed of each subject, we can still monitor the speed-related variations by using the goniometer. Based on the segment of Gait, we calculated the average duration of each Gait segment for each subject as shown in Figure 3. Independent-samples *t*-test was performed to compare the difference of average duration of Gait between subjects in CG and SG, and the results showed no significant difference (*p* = 0.693). With the exception of one subject (SG group, subject No.6), subjects in both CG and SG walked at a similar gait cycle (1.32 ± 0.103 s).

### 3.3. Data Analysis

#### 3.3.1. Non-Negative Matrix Factorization (NMF) Decomposition for Each Segment

The processed sEMG data for each segment were resampled to 100 samples to generate the sEMG signal matrix (a 4 × 100 matrix with 4 channels of sEMG and 100 sample points). Then the NMF algorithm was applied to these matrices. To optimize the number of synergies, the VAF were calculated for each matrix while changing the synergy number k from 1 to 4. Optimal k of each motion were set as the minimum number of synergies that makes the mean global value of VAF larger than 0.85 [41].

#### 3.3.2. Building Representative Muscle Synergy for Each Motion of Each Subject

For each subject, the synergy pattern matrices extracted from the EMG data of each motion were grouped and averaged across segments to generalize the representative muscle synergy matrix. In particular, the outlier matrices were removed from the averaging by the following steps: first, the similarities of each synergy pattern matrix with other matrices in the same group were calculated and then averaged. Afterwards, the distribution of all the average similarities were analyzed, and the matrices with average similarities that cannot satisfy the following equation were considered outliers.

(6) $$S_{i}-\bar{S}<3\delta ,$$

where, $S_{i}$ is the average similarity of the *i*-th matrix, $\bar{S}$ and δ are the mean and standard deviation of all the average similarities in the group. Out of all 385 synergy pattern matrices, 10 were considered outliers and were eliminated.

For each motion segment of each subject, the activation coefficient matrix is calculated by the method described in Section 2.1 under the premise of determining representative synergy pattern matrix. Afterwards, the activation coefficient matrices are averaged across motion segments to obtain the representative activation coefficient for each motion of each subject.

Finally, 2 × 11 × 3 (2 subject types, 11 subjects for each type and 3 motions) representative muscle synergy matrices and 2 × 11 × 3 representative activation coefficient matrices were generalized for the similarity analysis.

#### 3.3.3. Similarity Analysis

Analysis of intra-similarity of the muscle synergies in three lower limb motions within different subject groups (which were CG group, SG group and the combined group consisting of all subjects) were implemented, while the inter-similarity of muscle synergies between different subject groups were also calculated. Results of these analyses can reveal the differences in the muscle synergy of the thigh muscles during three motions between subjects with and without knee pathology, which may be helpful for the diagnosis of knee abnormal based on the muscle synergy analysis. In addition, similarities of muscle synergies between different motions were also analyzed for all subject groups, which may be helpful to establish a lower limb motion classifier based on muscle synergy analysis.

### 3.4. NMF-Based Classification of Lower Limb Motions with sEMG Data

#### 3.4.1. Segmentation

The processed sEMG data were divided into epochs by a moving window, which consists of 256 samples with an overlap of 64 samples [19]. Then each epoch of sEMG data were supplied to the NMF algorithm to extract the synergy pattern matrix $W=\left[w_{1}\cdots w_{k}\right]$ and the activation coefficient matrix $H={\left[h_{1}\cdots h_{k}\right]}^{T}$. Considering that the optimal synergy number k is different in different motions (2 in ST and STD, 3 in Gait, which are described in Section 4.1), the NMF algorithm was executed once for k = 2 and once for k = 3 to figure out the best solution.

#### 3.4.2. Feature Extraction

To facilitate the classification, features were extracted from W and H. Nonetheless, due to the random initialization in NMF algorithm, the order of the column vectors of W (synergy pattern vectors) and row vectors of H (activation coefficient vectors) are also random. To solve this problem, vectors were rearranged in the order of the corresponding activation ratios from largest to smallest. The activation ratio of the *i*-th synergy pattern vector as well as that of the *i*-th activation coefficient vector are calculated by the following equation:

(7) $$r_{i}=\frac{RMS\left(h_{i}\right)}{\sum_{i=1}^{k}RMS\left(h_{i}\right)},\;$$

Weights of corresponding muscles in the reordered synergy pattern vectors were selected as features, as $\left[w_{1}^{1},w_{1}^{2},w_{1}^{3},w_{1}^{4},\ldots ,w_{k}^{1},w_{k}^{2},w_{k}^{3},w_{k}^{4}\right]$; where $w_{i}^{j}$ represents the weight of *j*-th muscle of *i*-th synergy pattern vector. Moreover, a set of time-domain features were also generated from each reordered activation coefficient vector, which was a time series $h\left(t\right)$, *t* = 1,…,N, where N = 100. Features extracted from H and their computational formula are shown as follows:

- *RMS*.

(8) $$RMS=\sqrt{\frac{1}{N}\sum_{t=1}^{N}h{\left(t\right)}^{2}},$$

- Fourth-order auto-regressive model (*AR*).

(9) $$h\left(t\right)=-\sum_{i=1}^{4}a_{i}h\left(t-i\right)+e\left(t\right),$$

where, $a_{i}$ and $e\left(t\right)$ are the AR parameters and residual, respectively.

- Interquartile Range (*IQR*).

(10) $$IQR=Q_{3}-Q_{1},$$

where, $Q_{1}$ and $Q_{3}$ are the first and third quartile of h, respectively.

- Waveform Length (*WL*).

(11) $$WL=\sum_{t=1}^{N-1}\left|h\left(t+1\right)-h\left(t\right)\right|,$$

- Mean Absolute Value (*MAV*).

(12) $$MAV=\frac{1}{N}\sum_{t=1}^{N}\left|h\left(t\right)\right|,$$

#### 3.4.3. Feature Selection

Two feature combinations were tested both in the case of k = 2 and k = 3 in the present work. One of them contains only the features obtained from W (coarse feature sets, 8 features in the case of k = 2, 12 features in the case of k = 3), and the other contains all the features generated from W and H (fine feature sets, 8 + 2 × 8 = 24 features in the case of k = 2, while 12 + 3 × 8 = 36 features in the case of k = 3).

The Relief-F Index (RI) is used to evaluate the class separability of the features [42,43]. The Relief-F is an iterative algorithm that gives weight to each feature by calculating its correlation to all categories, with the higher the correlation with classification performance, the higher the weight. The update rule for Relief-F is:

(13) $$\begin{array}{l} RI\left(A\right)=RI\left(A\right)-\sum_{j=1}^{k}diff\left(A,R,H_{j}\right)/\left(mk\right)+\\ \sum_{C\notin class\left(R\right)}\left[\frac{p\left(C\right)}{1-p\left(class\left(R\right)\right)}\sum_{j=1}^{k}diff\left(A,R,M_{j,C}\right)\right]/\left(mk\right) \end{array}$$

where, $RI\left(A\right)$ represents the weight of feature *A*, before the iteration, all $RI\left(A\right)$ were initialized to 0. Moreover, m is the number of iterations and was set to equal the number of samples, *k* is the number of k-nearest neighbors, which was set to 1 in this work. $diff\left(A,B,C\right)$ means the distance between sample B and Sample C in the dimension of feature A, which is calculated as:

(14) $$diff\left(A,B,C\right)=\frac{\left|B\left[A\right]-C\left[A\right]\right|}{\max\left(A\right)-\min\left(A\right)},$$

This considered that the raw feature vectors in the fine feature sets were multidimensional data with redundant and irrelevant information. A two-stage feature selection method was introduced. First, RI of all features were calculated, and those with RI less than zero were eliminated. Then, the sequential forward selection (SFFS) algorithm was carried out to reduce the dimension of the retained feature sets [44,45]. In particular, the evaluation criterion of the SFFS algorithm was the classification performance of selected feature subsets with the random forests (RFS) classifier.

#### 3.4.4. Data Classification and Cross Validation

Note that the raw data were unbalanced between different classes, which may lead to classification bias towards the majority class by using the unbalanced raw data as training set directly. To overcome this barrier, a resampling method was applied. Specifically, the minority class was oversampled through the ADASYN algorithm and the majority class was undersampled by random sampling with replacement [46,47] while the resampling scale was set to the geometric mean of the scale of all classes.

The RFS algorithm was applied as the classifier. RFS, which was first proposed by Breiman [48], is an ensemble algorithm consisting of many decision tree-based classifiers. Compared with other classifiers like support vector machine (SVM) and K-nearest neighbor algorithm (KNN), it has high classification accuracy and calculation speed, as well as good anti-overfitting and anti-noise performance. The number of trees is an important parameter of RFS, increasing the number of trees results in a linear increase in running time and an increase in classification performance [49]. In the present study, we calculated the classification performance when the number of trees increased from 8 to 100, and the relationship curve between the number of trees and classification performance could be approximately fitted to a first-order rational curve, which means that there is an upper bound on the increase in performance. Considering the classification performance and operation efficiency of the algorithm, the number of trees was set to 30.

The 10-fold cross validation method was adopted to evaluate the classification performance. This required randomly splitting the NMF-based feature data set into 10 subsets, and repeating the validation method 10 times. For each time, one of the 10 subsets was selected as the testing set with the remaining 9 subsets as training set. The classification performance was determined as the average F-measure (FM) of all 10 folds. The FM is a classification performance evaluation metric obtained as the weighted harmonic average of precision and recall.

(15) $$P_{i}=\frac{n_{ii}}{\sum_{j=1}n_{ji}},$$

(16) $$R_{i}=\frac{n_{ii}}{\sum_{j=1}n_{ij}},$$

(17) $$FM_{i}=\frac{2P_{i}R_{i}}{P_{i}+R_{i}},$$

where, $n_{ij}$ represents the number of samples identified as class *j* and actually class *i*.

### 3.5. Diagnosis of Knee Pathology with NMF of sEMG Data

An additional pilot study was conducted to explore the feasibility of using NMF-based sEMG features in the diagnosis of knee pathology. Features used for diagnosing were extracted with the same methods described above, while the only difference was to change the multi-category label of different lower limb motions to the binary label of with or without knee pathology. Moreover, the methods described in Section 3.4 were also used to identify subjects with or without knee pathology.

### 3.6. Statistical Analysis

An independent sample *t*-test was used to check the difference in average duration of Gait between CG and SG. One-way analysis of variance (ANOVA) was applied to evaluate the intra-group and inter-group differences of similarities of muscle synergy in different subject groups or movement pairs. Wilcoxon signed-rank tests was utilized to analyze the influence of synergy number and feature sets on the classification performance. Results were considered significant for *p* < 0.05.

## 4. Results

### 4.1. Optimal Number of Muscle Synergies

**Figure 4:**
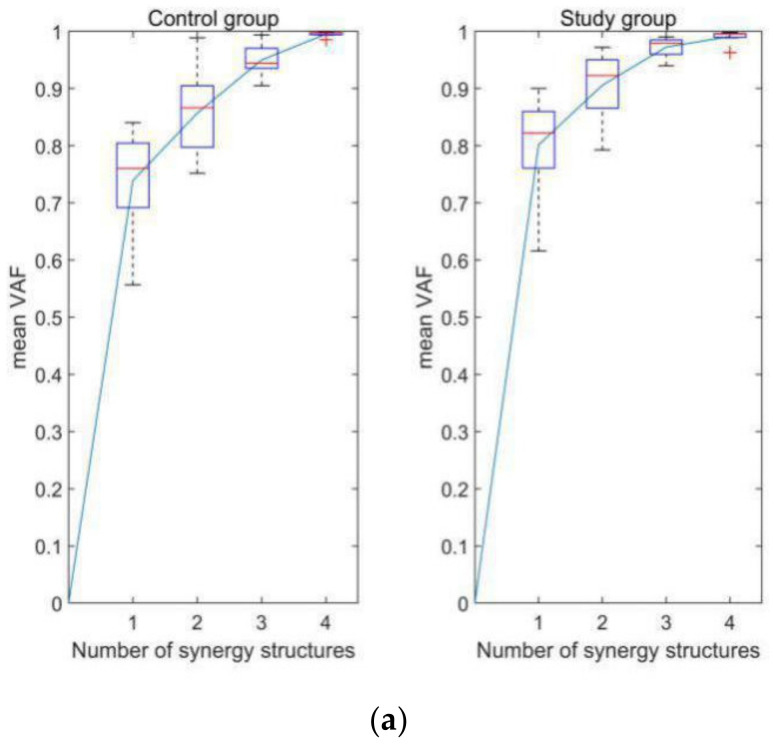

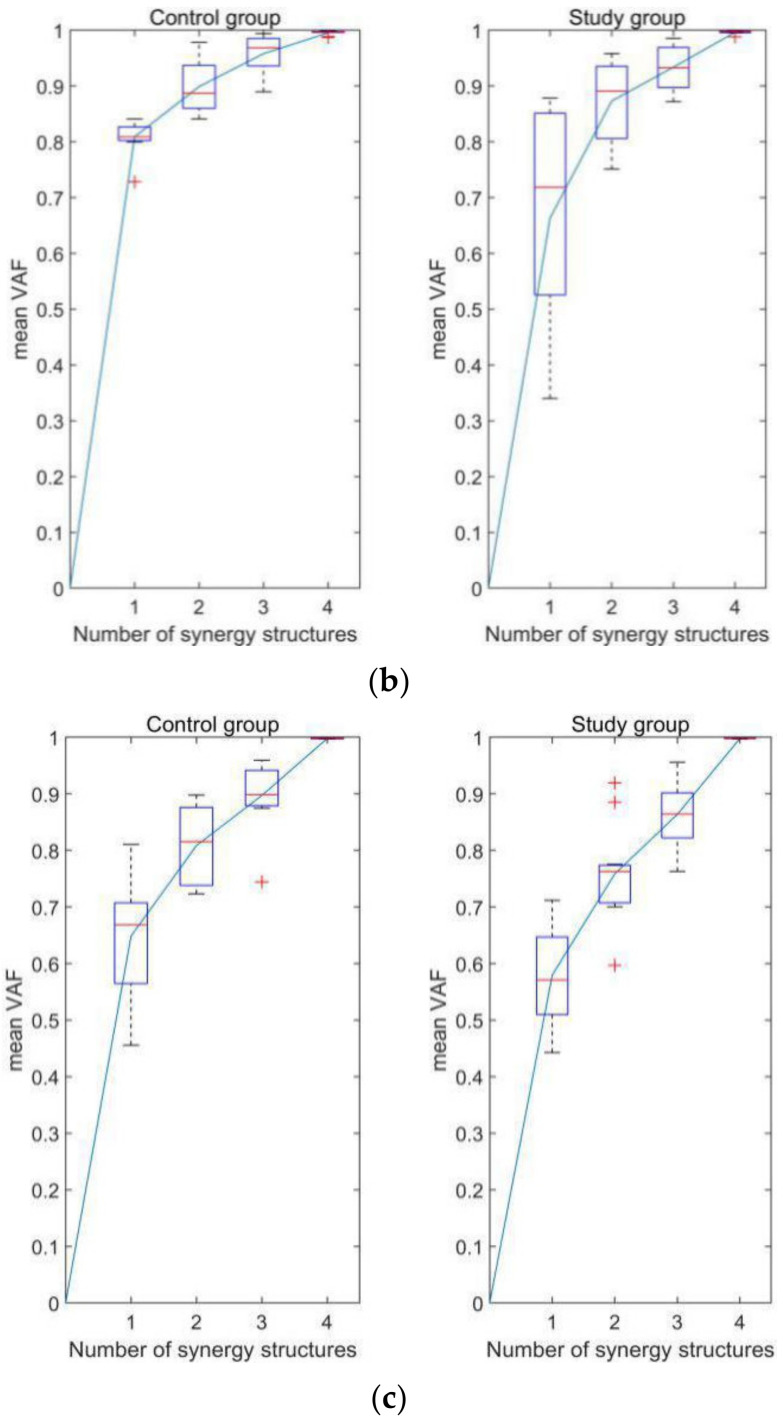
Relationship between the number of muscle synergy structures and the mean VAF in different groups when performing different lower limb motions. (**a**) STD, (**b**) ST, and (**c**) Gait. The ‘+’ in the figure represents the outliers.

The mean VAF of CG group and SG group performing different motions when k equals from 1 to 3 are shown in Table 1. Figure 4 shows the mean VAF as functions of the numbers of muscle synergies in different subject groups when performing different lower limb motions. According to the criteria described in Section 3.3.1, the minimum number of synergies that makes the mean VAF larger than 0.85 is 2 for STD and ST, while 3 for Gait. Therefore, these parameters were used in subsequent studies.

**Figure 5:**
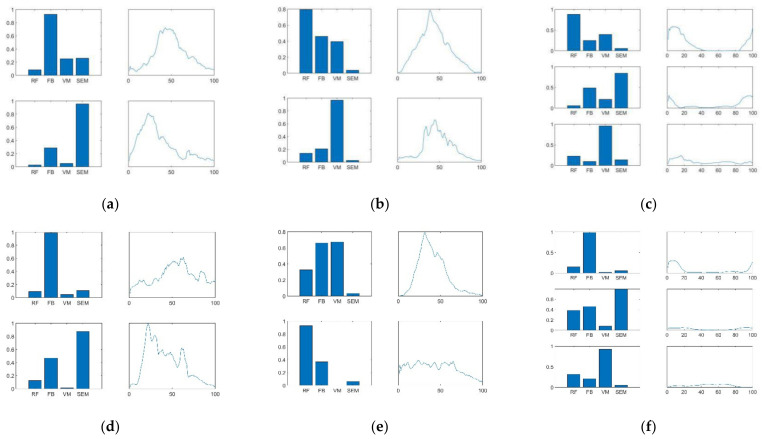
Typical examples of the muscle synergy pattern and the corresponding activation coefficient with the optimal number of muscle synergies in CG (subject No.10, denote as CG10) and SG (subject No.11, denote as SG11). The muscle synergy and its corresponding activation coefficient of each subgraph from top to bottom is arranged from large to small according to its activation ratio described in Section 3.4.2. (**a**) The STD of CG10, (**b**) the ST of CG10, (**c**) the Gait of CG10, (**d**) the STD of SG11, (**e**) the ST of SG11, (**f**) the Gait of SG11.

Figure 5 shows typical examples (CG group, subject No.10 and SG group, subject No.11) of the muscle synergy pattern and the corresponding activation coefficient with the optimal number of muscle synergies. The synergy patterns among thigh muscles extracted from different lower limb motions are different. Take the subject CG10 cited in Figure 5 as an example, FB and SEM play the main role during STD, while RF and VM are primarily activated during ST. The activation ratios of the two muscle synergies are comparable during STD and ST. However, all four muscles are activated to varying degrees during Gait. The first two muscle synergies produce most of the contributions with a combined activation ratio of 79%. According to the first two synergies, for subject CG10, RF and SEM provide the main contribution during Gait and the main period of activation of the thigh muscle synergies is the support phase.

### 4.2. Muscle Synergy Similarities

**Figure 6:**
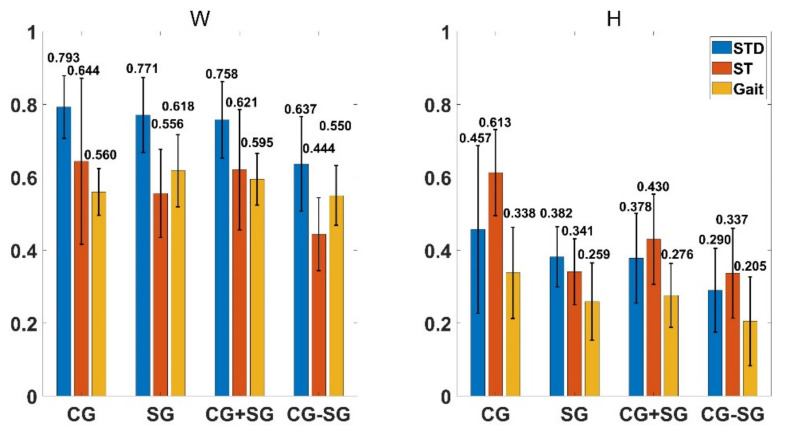
Intra-similarity of different subject groups (CG, SG and the combined group CG + SG) and the inter-similarity between CG group and SG group (CG-SG) when performing three different motions (STD, ST and Gait). The numbers on the bar represent the corresponding average similarity coefficient.

**Figure 7:**
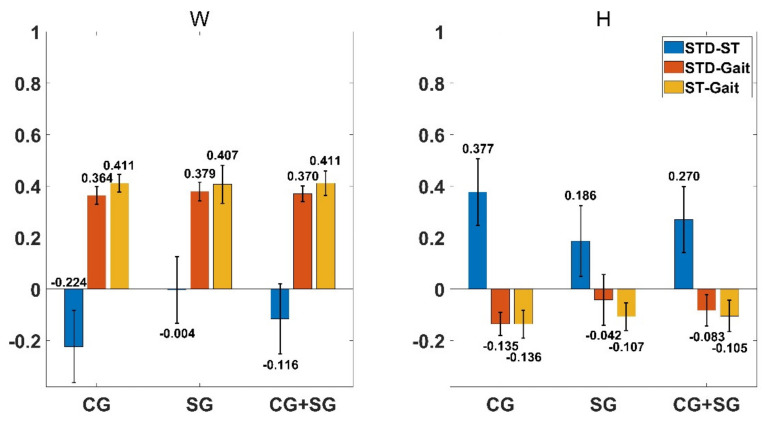
Inter-similarity between any two of the three motions (STD, ST and Gait) performed of different subject groups (CG, SG and the combined group CG + SG). The numbers on the bar represent the corresponding average similarity coefficient.

The intra-similarities of muscle synergy pattern and activation coefficient in each subject group when performing different motions are shown in Figure 6. The muscle synergy pattern extracted from each subject group when performing STD has high degree of intra-similarity. While, the intra-similarity of muscle synergy pattern when performing ST and Gait are moderate. Moreover, the intra-similarities of activation coefficient in each group are much lower than that of the synergy pattern. The inter-similarity of muscle synergy between the CG group and SG group is also shown in Figure 6. For muscle synergy pattern, the inter-similarity is at a moderate level, while for activation coefficient, the inter-similarity is much lower. Figure 7 shows the inter-similarity between different motions (three different motion pairs, which are STD against ST, STD against Gait and ST against Gait) in different groups. The inter-similarity of synergy pattern between STD and ST was significantly lower than that between Gait and each of the other two motions (*p* < 0.05, one-way ANOVA), while the opposite is true in that of activation coefficient (*p* < 0.05, one-way ANOVA). Besides, in both CG and SG, the similarities of synergy pattern and activation coefficient when performing the same lower limb motion were significantly higher than that when performing different motions (*p* < 0.05, one-way ANOVA).

### 4.3. Lower Limb Motions Classification

**Figure 8:**
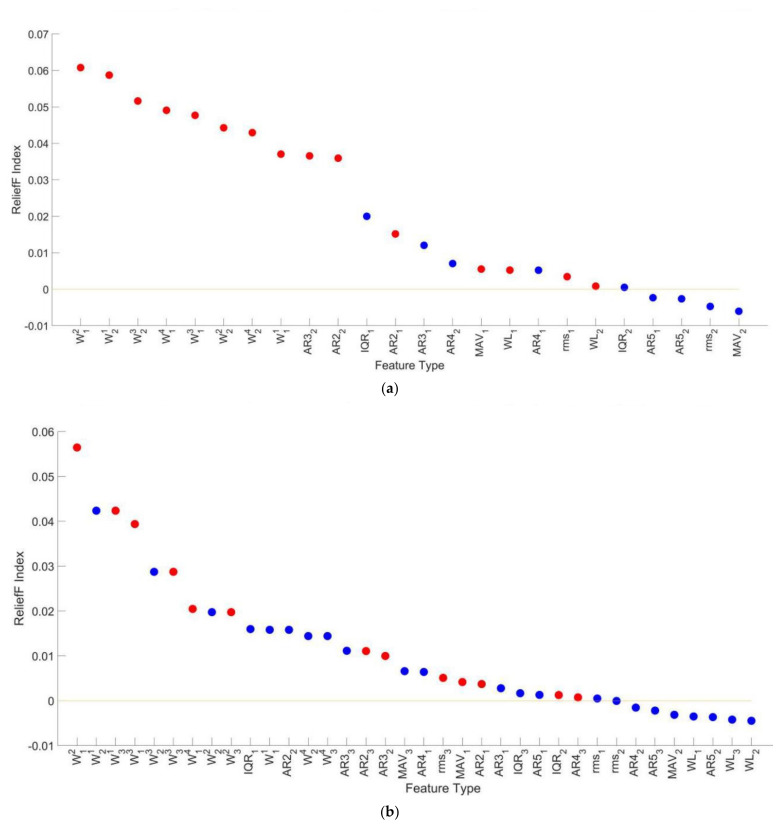
The Relief F Index of all features in the fine feature sets and features (the red scatters) selected by the feature selection method in different synergy numbers. (**a**) k = 2 and (**b**) k = 3. The blue scatters represent features that are not selected by the feature selection method.

Figure 8a shows the RI of all features when k = 2 in the decreasing order. 4 features ($AR5_{1}$, $AR5_{2}$, $rms_{1}$ and $MAV_{1}$, where the subscript *i* in feature $F_{i}$ means the *i*-th synergy pattern vector or activation coefficient vector) with RI less than zero were eliminated in the first step of the feature selection method described in Section 3.4.3. Moreover, the red scatters in Figure 8a represents all the features obtained by the feature selection method. To be specific, 15 features were selected, which were all the 8 features in the coarse feature sets, $rms_{1}$, $AR2_{1}$, $WL_{1}$, $MAV_{1}$, $AR2_{2}$, $AR3_{2}$ and $WL_{2}$. Moreover, the class separability of features when k = 3 is shown in Figure 8b. Therein, 8 features ($rms_{2}$, $AR4_{2}$, $AR5_{3}$, $MAV_{2}$, $WL_{1}$, $AR5_{2}$, $WL_{3}$ and $WL_{2}$) were eliminated and 13 features ($W_{1}{}^{2}$, $W_{1}{}^{3}$, $W_{1}{}^{4}$, $W_{3}{}^{1}$, $W_{3}{}^{2}$, $W_{3}{}^{3}$, $AR2_{1}$, $MAV_{1}$, $AR3_{2}$, $IQR_{2}$, $rms_{3}$, $AR2_{3}$ and $AR4_{3}$) were selected.

The classification performance of different feature sets in three subject groups with two kinds of synergy number are listed in Table 2. Mean FM of the coarse feature sets is the smallest among all three feature sets, while the classification performance of the fine feature sets and selected feature sets are comparable. Significance probability of Wilcoxon signed-rank tests on mean FM of classification on feature sets when k = 2 and k = 3 is below 0.05, and FM of classification on feature sets when k = 2 is higher than that when k = 3 in all conditions. Therefore, subsequent studies on motion identification were all carried out under the condition of k = 2.

**Figure 9:**
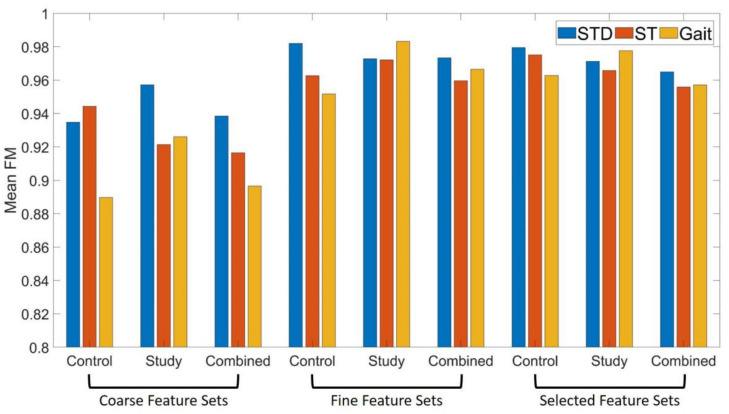
Mean F-measure per lower limb motion for different subject groups and feature sets.

Figure 9 shows the performance of motion classification with all feature sets extracted from subjects of different groups performing three lower limb motions when synergy number is set to 2. Motion classification performance with fine feature sets and selected feature sets are significantly higher than that with coarse feature sets (*p* < 0.05, Wilcoxon signed-rank tests).

### 4.4. Knee Pathology Identification

**Figure 10:**
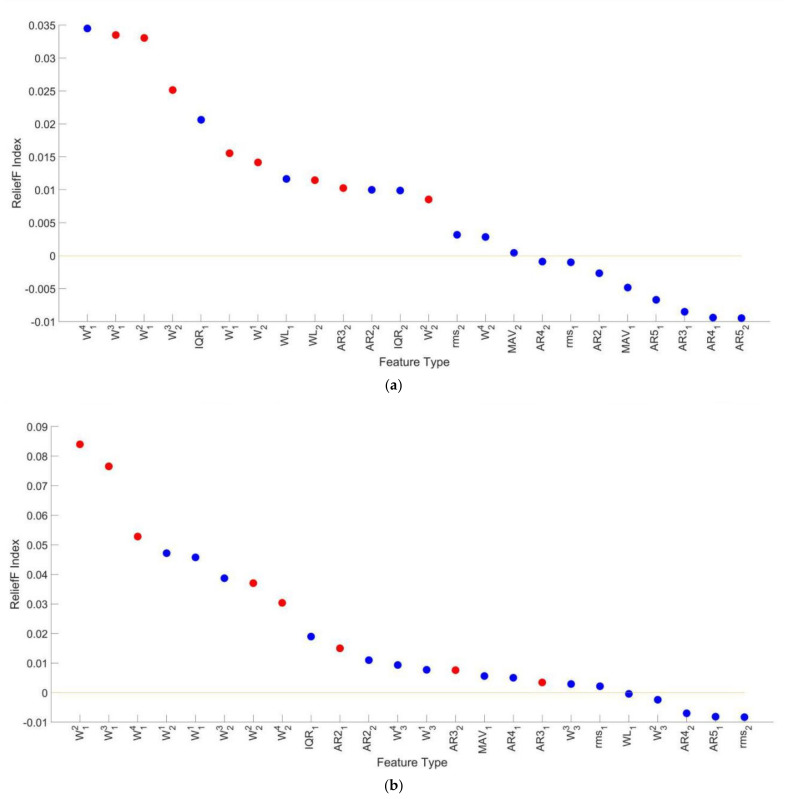

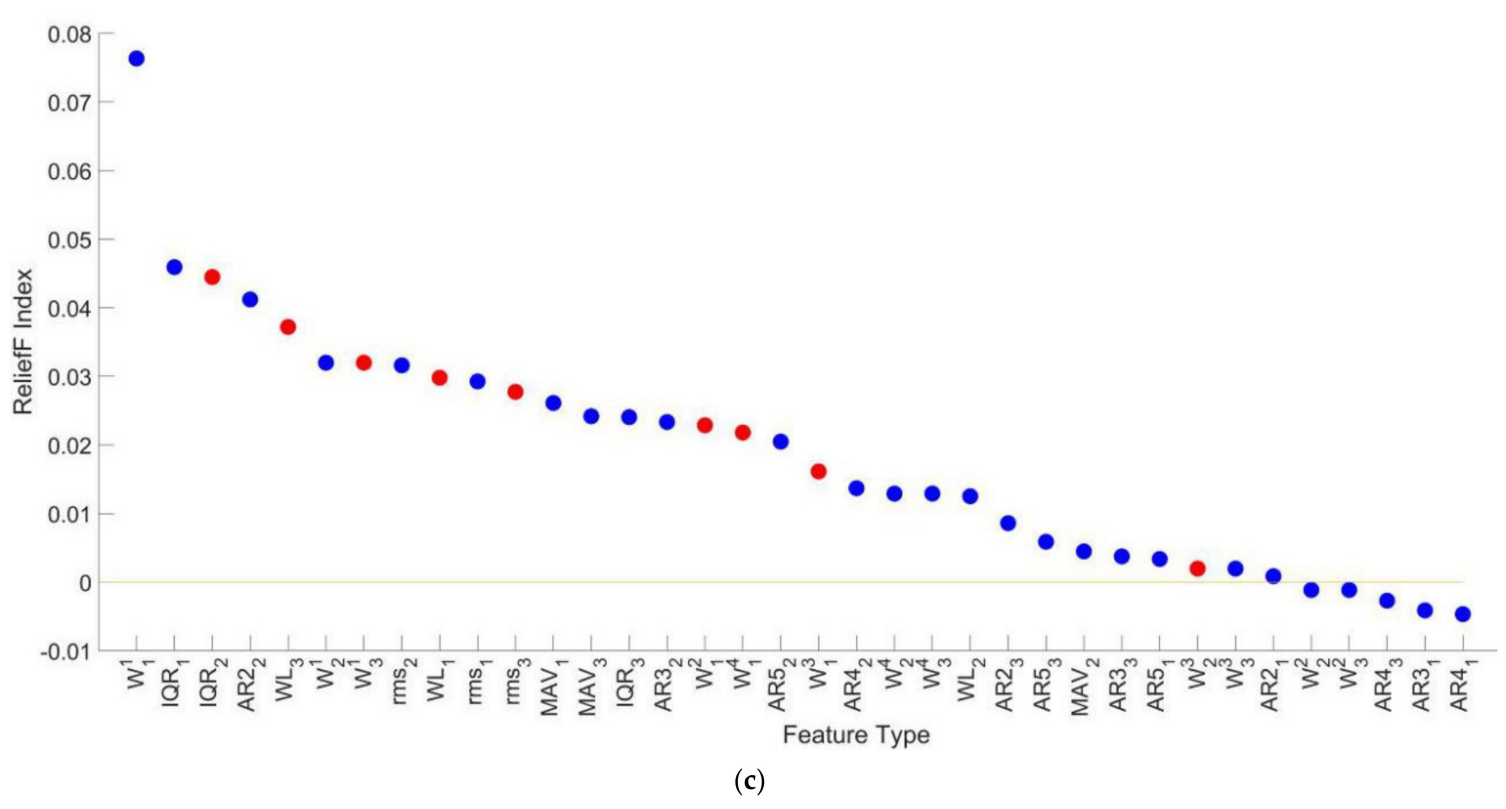
The ReliefF Index of all features in the fine feature sets and features (the red scatters) selected by the feature selection method when performing different motions. (**a**) STD, (**b**) ST and (**c**) Gait. The blue scatters represent features that are not selected by the feature selection method.

Results of feature selection for NMF based knee pathology identification are shown in Figure 10. Figure 10a reveals that for performing STD, 8 features ($AR4_{2}$, $rms_{1}$, $AR2_{1}$, $MAV_{1}$, $AR5_{1}$, $AR3_{1}$, $AR4_{1}$, $AR5_{2}$) with RI lower than zero were eliminated in the first step of feature selection and additional 8 features ($W_{1}^{1}$, $W_{1}^{2}$, $W_{1}^{3}$, $W_{2}^{1}$, $W_{2}^{2}$, $W_{2}^{3}$, $WL_{2}$, $AR3_{2}$) were selected in the second step. In Figure 10b, 5 features ($WL_{1}$, $W_{3}^{2}$, $AR4_{2}$, $AR5_{1}$ and $rms_{2}$) were eliminated in the first step and 8 features ($W_{1}^{2}$, $W_{1}^{3}$, $W_{1}^{4}$, $W_{2}^{2}$, $W_{2}^{4}$, $AR2_{1}$, $AR3_{2}$, $AR3_{1}$) were selected in the second step for performing ST. While, for performing Gait, Figure 10c shows that 5 features ($W_{2}^{2}$, $W_{3}^{2}$, $AR4_{3}$, $AR3_{1}$, $AR4_{1}$) were eliminated and 9 features ($W_{1}^{2}$, $W_{1}^{3}$, $W_{1}^{4}$, $W_{2}^{3}$, $W_{3}^{1}$, $IQR_{2}$, $WL_{3}$, $WL_{1}$, $rms_{3}$) were selected at the end of the feature selection.

**Figure 11:**
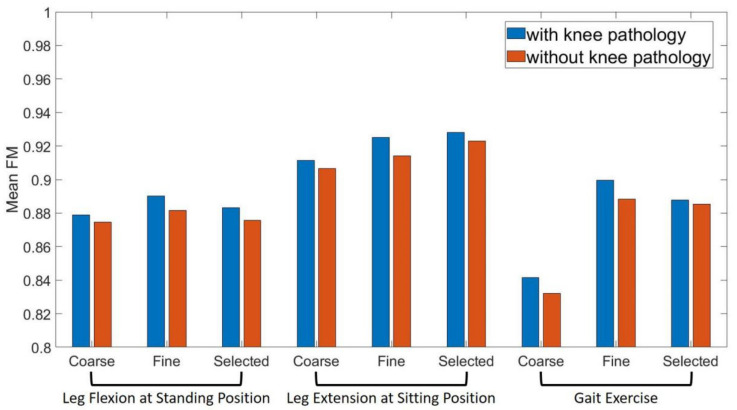
Mean F-measure of identifying subjects with or without knee pathology at different feature sets and lower limb motions.

The classification performance of identifying subjects with or without knee pathology when performing different lower limb motions by using different feature sets are shown in Figure 11. The mean FMs of negative subjects (subjects without knee pathology) and positive subjects (subjects with knee pathology) are 0.895 and 0.887, respectively. Moreover, mean FMs for knee pathology identification based on sEMG synergy information when performing STD, ST and Gait are 0.875, 0.918 and 0.880, respectively. Mean FMs obtained by using coarse feature sets, fine feature sets and selected feature sets are 0.867, 0.903 and 0.902, respectively.

All the data analysis and classification algorithms were implemented in Matlab R2018a on a 3.0 GHz Inter(R) Core_(TM) i5 processor with 8 GB RAM. Mean time for NMF of a segment of four channel sEMG data was 119 ms, while time for feature extraction was 11 ms for selected feature sets and 17 ms for fine feature sets. Additionally, the training time of RFS classifier with coarse feature sets, fine feature sets and selected feature sets were 3.704 s, 5.771 s and 3.825 s, respectively. While, the prediction speed for the three feature sets were approximately 14,000 observation per second (obs/s), 11,000 obs/s and 13,000 obs/s, respectively.

## 5. Discussion

The present work studies the muscle synergy of thigh muscles during three lower limb motions. According to the muscle synergy hypothesis, muscle synergy is the minimum unit for CNS to recruit skeletal muscles to perform various movements, and the neuromuscular control of complex and natural motions can be regard as linear combination of several muscle synergies with different functions. STD, ST and Gait reveal the muscle synergy in knee flexion, extension and hybrid motion, respectively. Although synergy number in Gait is larger than those in STD and ST, results of the present work show that a small amount of muscle synergy can be used to reconstruct the sEMG signals of the thigh muscles in the three motions. The reason for the difference in synergy number between Gait and the other two motions may be that the former involves motion of bilateral multi-joints with multi-degree of freedom, while the latter mainly involves a single degree of freedom of knee flexion or extension. In fact, only one synergy pattern is required to reconstruct the STD or ST for some subjects, as shown in Figure 4, while it cannot be achieved in Gait. Extending the sEMG channels to the shank and contralateral lower limb can solve this problem. For example, a study by Hikaru Yokoyama [50] showed that the sEMG information of 16 ipsilateral lower limb muscles could be reconstructed by only one or two synergy patterns during walking at different speeds. Results of muscle synergy analysis of the present study provide new evidence for the muscle synergy hypothesis in the neuromuscular control of knee movements.

### 5.1. Muscle Synergy Differences between Different Subject Groups or Different Motions

By analyzing the similarities between subjects in performing specific lower limb motions, the results of the present work showed the consistency in synergy pattern across subjects within the same motion. In Figure 6, the degree of intra-similarity of muscle synergy pattern within each subject group has high level in STD as well as medium level in ST and Gait, even in the worst case the intra-similarity coefficient of synergy pattern is still 0.556. However, such consistency did not appear in activation coefficient under the same conditions mentioned above. These findings suggest that the CNS tends to recruit similar muscle synergy patterns when performing the same lower limb motions in different subjects, and the subjects’ personalities are mainly defined by the difference of the activation sequences of these patterns.

Figure 7 shows an opposite results on the differences of inter-similarity between different motion pairs in synergy pattern and activation coefficient. The reason for this phenomenon is that the CNS mainly recruit the single flexion or extension function of the knee in STD or ST, while both functions are recruited in Gait. Nonetheless, the degree of inter-similarity between each motion’s pair was low either in synergy pattern or activation coefficient.

According to the results of Figure 6 and Figure 7, the intra-similarity of muscle synergy within same motions is significantly higher than the inter-similarity between different motions, which indicates that it is feasible to use the features extracted from the muscle synergy matrices for lower limb motion recognition. In particular, although the inter-similarity of muscle synergy across subject groups was lower than the intra-similarity within each group when performing different motions (0.114 less on average for synergy pattern and 0.109 less on average for activation coefficient), it was still much greater than the inter-similarity between different motions (0.245 higher on average for synergy pattern and 0.117 higher on average for activation coefficient), which suggests that the lower limb motion recognition based on muscle synergy may have good consistency in both healthy subjects and subjects with knee pathology. Moreover, differences between the inter-similarity across subject groups and the intra-similarity within each subject group also indicated that it is possible to use muscle synergy information to distinguish subjects with or without knee pathology.

To date, several studies have analyzed the similarities of muscle synergy during different movements in different condition. For example, Akira Saito et al. [25,38] proved that the muscle synergies extracted from lower limb muscles are consistent between different walking conditions, such as ground inclination angle. However, this consistency changes with significant differences in speed, Yokoyama et al. [50] found that muscle synergies are different while walking and running. Ortega-Auriol et al. [51] studied the adaptions of muscle synergies to the development of fatigue and found that synergy patterns were conserved with fatigue, but activation coefficients decreased. When discussing the effects of neuromuscular disease, Francesca Lunardini et al. [52] found that despite children with dystonia presenting abnormal kinematics of the writing outcome, they still tend to have similar number and structure of the synergy vectors compared with age-matched controls during the execution of writing tasks. In a study that explored a muscle synergy-based method to assess the upper limb motor dysfunction of cerebral palsy children, Lu Tang et al. [30] analyzed the similarities of muscle synergy when performing three similar upper limb motion tasks between the patients and controls. They found many abnormal synergy patterns specific to the patients and developed seven muscle synergy based upper limb assessment metrics. These studies suggest the consistency and specificity of muscle synergies between different movements or different motion conditions (speed, fatigue and disease for example), which is similar to our similarity analysis of the muscle synergy of knee related muscles in different subject groups performing different movements.

### 5.2. Performance of Muscle Synergy-Based Lower Limb Motion Classifier

Several works have been undertaken by other researchers based on the dataset we used. For motion recognition, Marcelo Herrera-González [40] developed an MP-ANN classifier to recognize the lower limb motions by using the frequency domain and wavelet-based time-frequency domain features extracted from raw sEMG and a goniometer. Accuracy of identifying STD, ST and Gait in this work were 0.92, 0.94 and 0.88 on average, respectively. Although the classification performance was good, this work lacked a description of the classification performance in different subject groups. Furthermore, the high accuracy was obtained by the fusion of two heterogeneous sensors. Accordingly, the accuracy dropped to 0.82 when only sEMG was used. Building a robust, responsive and concise motion classifier with only sEMG information is useful in clinical application. However, Naik [19] et al. found that the classification performance is low by building a classifier with features extracted from raw sEMG segments. To solve this problem, they designed an ICA-EBM-based sEMG classification scheme by building an LDA classifier with time-domain features and fractal dimension extracting from the source estimates obtained by decomposing the multi-channel sEMG data with the ICA-EBM algorithm. The average classification accuracy in CG and SG of that work were 0.961 and 0.862, respectively. Note that the classification performance in SG was much lower than that in CG, and the data segments were obtained after the ICA-EBM algorithm had been adopted to the whole sEMG dataset, which may be difficult in real-time applications.

In the present work, the lower limb motion recognition were realized by building a RFS classifier using features extracted from NMF of the multi-channel sEMG data. Table 2 shows that the classification performance in CG and that in SG is comparable, which meets the analysis on the muscle synergy similarity mentioned above. Note that the mean FMs of motion classification with all three feature sets are higher than 0.9, and higher than 0.96 for the feature sets extracted from both the synergy pattern matrix and corresponding activation coefficient matrix as well as the reduction version of this feature set. These results indicate that the NMF-based sEMG lower limb motion recognition has good and consistent performance among subjects with and without knee pathology.

Motion classification performance with fine feature sets and selected feature sets are significantly improved compared with that with coarse feature sets, which suggests that the muscle synergy activation sequence contains additional useful information that the muscle synergy pattern dose not. In addition, motion recognition by using selected feature sets can improve the training and testing speed compared with by using all the features, while it can still achieve good and consistence classification performance compared with by using features only extracted from the synergy pattern. Therefore, the NMF-based sEMG lower limb motion recognition with the selected feature sets has great potential in real-time applications, especially in the case of limited number of sensors required, such as intelligent prosthetic control for patients with calf cutoff.

### 5.3. Muscle Synergy Based Classification Is a Potential Method for Knee Pathology Diagnosis

For knee pathology diagnosis, Majid Janidarmian et al. [53] investigated eight different classification techniques with a set of time-domain features extracted from sEMG and goniometer data of the same dataset we used. The best accuracy was 0.972 by using the bagged decision tree in this work. Note that the high classification performance were achieved by using the fusion information of sEMG and a goniometer, and the slide window size was 5.5 s. Ertugrul et al. [54] built an adaptive local binary pattern (ALBP) algorithm to detect hidden patterns of sEMG signals, which can express the illness. The ALBP was adopted in the dataset we used, and achieved an accuracy of 0.85 for distinguishing subjects with and without pathology.

In the present work, the identification performance for knee pathology diagnosis when performing ST was better than that when performing the other two motions. For subjects with knee pathology, it is a challenging task to keep one leg supported while flexing the other leg. Compared with healthy people, subjects with knee pathology may need some compensatory muscle activation to overcome the damage caused, especially in a difficult task such as ST. This compensatory muscle activation can lead to changes in the muscle synergy pattern, which is evidenced by the results in Figure 6 that the inter-group similarity of synergy pattern when performing ST was significantly lower than that when performing the other two motions (*p* < 0.05, one-way ANOVA). Additionally, similar to the lower limb motion recognition, the selected feature sets is recommended in knee pathology identification due to its good classification performance and high speed.

Performance of knee pathology diagnosis can be improved by combining the sEMG information of subjects in various types of motion. Nonetheless, it must be remembered that the data used in the present work came from open source datasets, and the temporal relationships between different types of motions performed by the same subject are lacking, which makes it difficult to establish a fusion classifier based on multi-motion information. Therefore, only the diagnostic models of subjects performing each motion were established respectively in the present study. Besides, the knee pathology of subjects in SG was diverse and varying in number, which may also reduce the classification performance. However, despite these problems, the muscle synergy-based knee pathology classifier still achieved a good performance and showed the potential to assist in clinical applications, not only in the field of knee pathology diagnosis, but can also help to carry out targeted rehabilitation training for neuromuscular control abnormality of knee muscles.

### 5.4. Limitation and Future Work

This paper conducted a preliminary study on the muscle synergy of knee-related muscles of subjects with and without knee pathology when performing three lower limb motions. Based on this, a muscle synergy based lower limb motion recognition framework and a knee pathology diagnosis method were proposed. Despite some interesting achievements, there are still some limitations that need to be improved in future work.

Firstly, only 11 healthy adult male subjects and 11 adult male patients with knee pathology were involved in this study. The small number of subjects and incomplete demographic distribution, and lack of female subjects, for example, may limit the statistical results. Thus, more subjects with different kinds of demographic information should be recruited in future work. Secondly, the open datasets we used included only four major thigh muscles, which may limit our understanding of neuromuscular control strategies in knee movements, especially in complex motions such as walking. More muscles should be considered in the future. Finally, patients in this work had three different knee pathologies, which affects the accuracy and specificity of the diagnostic classifier, so there is still a lot of work to be done in the clinical application of muscle synergy-based knee pathology diagnosis. Future work should focus on designing continuous and complete test methods, collecting more data to train the hybrid model, and proposing the assessment metrics for specific knee pathology.

## 6. Conclusions

In the present study, muscle synergy analysis of three different lower limb motions was conducted in subjects with and without knee pathology. Intra-group similarity of synergy pattern when performing each motion was found to be at a medium-to-high level, while inter-subject group similarity was lower than intra-group similarity. By contrast, the activation coefficient similarity of subject groups was much lower, but the relation between intra-subject group similarity and inter-subject group similarity in the activation coefficient was the same as that in the synergy pattern. Moreover, the inter-similarity of either the synergy pattern or activation coefficient between each two motions is generally at a low level. The studies on muscle synergy similarity revealed the feasibility of identifying lower limb motions or distinguishing subjects with or without knee pathology by using NMF of sEMG signals.

In order to improve the classification performance of NMF-based lower limb motion recognition, a two-stage feature selection method was introduced. Results show that the RFS classifier by using the selected feature sets containing entries of synergy pattern matrices and time-domain features of activation coefficient sequences can achieve good classification performance and high execution speed. A similar study was carried out on the diagnosis of knee pathology with NMF of sEMG signals. The mean FM of that was highest with selected feature sets extracted from NMF of sEMG signals when subjects were performing ST.

The proposed methods can be serve as a promising approach to develop a tool for quantifying the underlying neuromuscular mechanisms involved in the execution of lower limb motions for patients, which can be used for knee pathology diagnosis, motion recognition and rehabilitation.

## Figures and Tables

**Table 1:** The mean variability accounted for (VAF) of Control Group (CG) and Study Group (SG) performing different motions when k equals different values.

Motion	Number of Synergy	Control Group	Study Group
Leg flexion at standing position (STD)	1	0.7388	0.8015
	2	0.8570	0.9058
	3	0.9500	0.9725
Leg extension at sitting position (ST)	1	0.8088	0.6633
	2	0.8989	0.8730
	3	0.9580	0.9338
Gait exercise (Gait)	1	0.6486	0.5797
	2	0.8085	0.7586
	3	0.8973	0.8639

**Table 2:** Mean F-measure of different subject groups and different feature sets with different synergy number.

Feature Sets	Subject Group	k = 2	k = 3
Coarse Feature Sets	Control Group	0.920	0.902
	Study Group	0.938	0.910
	Combined Group	0.913	0.897
Fine Feature Sets	Control Group	0.968	0.955
	Study Group	0.973	0.958
	Combined Group	0.962	0.952
Selected Feature Sets	Control Group	0.964	0.944
	Study Group	0.973	0.949
	Combined Group	0.960	0.943
